# Complex In Vivo Motion of the Bovine Tail Provides Unique Insights Into Intervertebral Disc Adaptation

**DOI:** 10.1002/jsp2.70084

**Published:** 2025-06-17

**Authors:** Arthur J. Michalek, Isabelle M. Wood, Daniela Gonzalez Carranza, Lindsay Ferlito

**Affiliations:** ^1^ Department of Mechanical & Aerospace Engineering Clarkson University Potsdam New York USA; ^2^ Department of Chemistry & Biochemistry Clarkson University Potsdam New York USA; ^3^ College of Agriculture & Life Sciences, Cornell University Ithaca New York USA

**Keywords:** annulus fibrosus, collagen fiber crimp, motion tracking, repetitive motion

## Abstract

**Introduction:**

The intervertebral disc (IVD) of the bovine tail is a commonly used research analogue for the human disc at the organ, tissue, and cellular levels. While these tails are subjected to thousands of dynamic motion events daily, little is known about how these motions might induce tissue remodeling, particularly in the outer annulus fibrosus (AF) of IVDs connecting adjacent vertebrae. This study hypothesized that despite the similarities in geometry and biochemical composition of IVDs in the bovine tail, level‐wise variations in repetitive in‐vivo motion would be associated with tissue level adaptations.

**Methods:**

In‐vivo active range of motion (RoM) was measured by placing inertial measurement unit sensors on the tails of adult cows and using a multi‐segment rigid body model to calculate level‐wise flexion‐extension and lateral bending angles. Level‐wise passive RoM was measured from cadaveric adult bovine tails in flexion, extension, and lateral bending with skin and muscles removed. IVDs were extracted for measurement of height, diameters, AF radial thicknesses, and AF fiber crimp periods.

**Results:**

In‐vivo joint RoM was found to vary drastically by level, largely due to a prominent second order mode with inflection point at the fourth joint. Joint levels near this inflection point were found to have the highest passive RoMs. In the proximal tail, decreased RoM was associated with an increased fiber crimp period in the outer AF, while in the distal tail it was associated with increased AF thickness.

**Discussion:**

Taken together, these findings suggest that IVDs in the bovine tail respond to repeated complex dynamic motions through a process of adaptation at the mesoscale (AF thickening during growth) and microscale (residual strain accumulation in the mature state). The bovine tail thus provides a powerful tool for modeling how the human lumbar intervertebral disc may remodel in response to changes in exposure to repetitive motions.

## Graphical abstract

The bovine tail experiences complex dynamic motions in vivo, resulting in varying amplitudes of lateral bending and flexion‐extension between levels. Joints exposed to the lowest motion amplitudes in vivo have the highest ex vivo passive ranges of motion. Decreased range of motion is associated with trends in intervertebral disc geometry and microstructure along the tail.

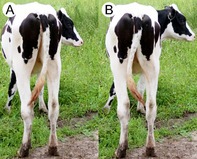

## 1 Introduction

Adjacent vertebrae in the spine are connected by intervertebral discs (IVDs), degeneration of which is a prominent cause of low back pain. The disc consists of an outer annulus fibrosus (AF) made up predominantly of concentric layers of collagen fibers oriented in alternating right‐ and left‐handed helices and an isotropic, proteoglycan‐rich, inner nucleus pulposus (NP). While the lamellar structure of the AF is established during growth [1, 2] cells of mature IVDs have been shown to express both anabolic and catabolic genes in response to various joint‐scale mechanical stimuli [3, 4, 5, 6, 7], suggesting remodeling in order to maintain optimal strain levels. Localized remodeling stimulated by gradients of strain throughout the disc has been suggested as a driver of residual strain accumulation in the outer AF of the bovine caudal disc [8]. While organ‐scale mechanical adaptation of the disc to loading is difficult to study due to the disc's slow metabolic rate, static bending of rat tail joints has been shown to result in increased bending stiffness along with increased AF collagen fiber crimp period [9].

A commonly used model in IVD research is the bovine caudal disc. This is an attractive analog for the human lumbar disc due to its similar structure and composition [10] along with similar size but simpler geometry [11]. Bovine tail discs exhibit low interspecimen variability and, as they are usually obtained from healthy animals at earliest skeletal maturity (typically between 18 and 24 months), minimal age‐related degeneration. The bovine caudal disc model has been employed across a wide range of applications. In biological studies, it has been employed as a cell source [12, 13], organ culture model [4, 5, 14, 15], and as a biomimetic scaffold [16]. In mechanical studies, the bovine caudal disc has been used from the tissue [17, 18, 19] to whole motion segment scales [20, 21, 22, 23, 24]. Despite this history of use as a model system in IVD research, in vivo mechanics of the bovine tail are sparsely studied.

**FIGURE 1:**
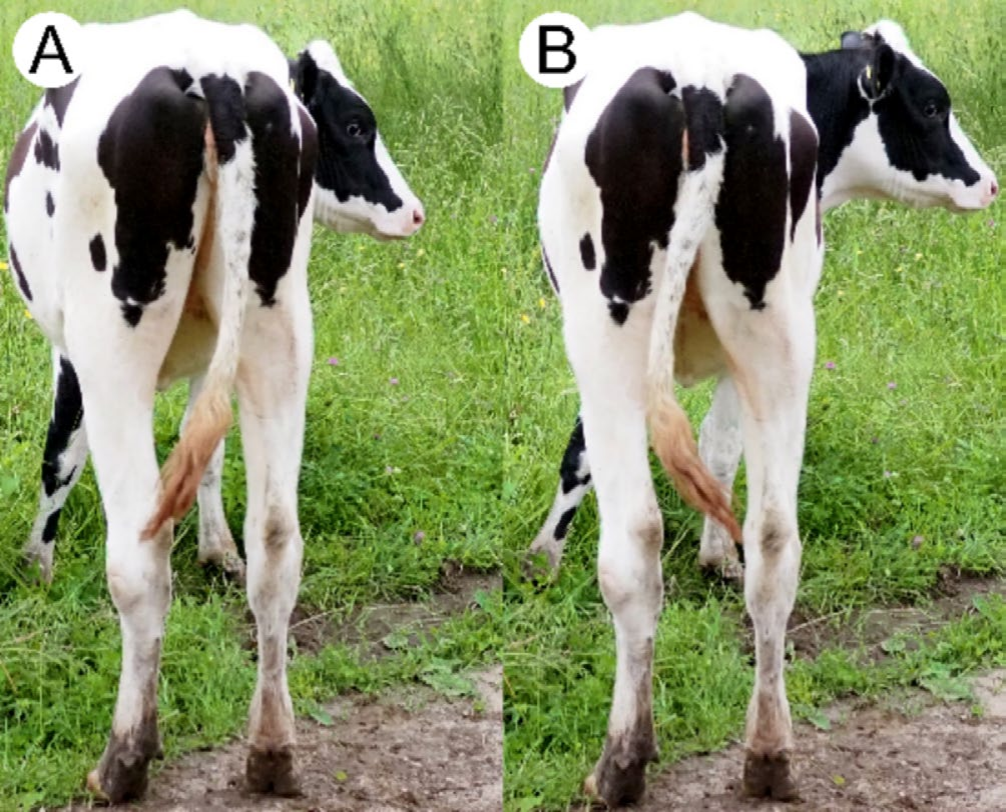
Examples of first (A) and second (B) mode lateral bends in the bovine tail.

Tails serve a number of functional purposes across the animal kingdom. Many species use tail movements as a mode of communication [25, 26, 27]. Animal tails have been found to provide stabilization during dynamic movement in species ranging from lizards [28, 29, 30] to primates [31]. In large mammals, the tail is frequently employed as a fly swatter [32]. This function is of particular interest due to its high frequency, with as many as 36 tail swishing events per minute having been previously observed in cows [33]. Dynamics of this functional tail motion and the resulting impact on joint‐level tissue remodeling have been sparsely studied. Prior study of the motion of mammalian tails has employed either a double pendulum model with a single oscillatory mode [32], or a continuous elastic beam with a constant radius of curvature [34]. However, visual observations of bovine tail motion suggest that a second mode, in which the tail takes on an “S” shape, may be frequently experienced (Figure 1).

Movement of the bovine tail is accomplished through contraction of the coccygeus muscles at the base of the tail, along with a series of intrinsic muscles connecting adjacent vertebrae within the tail providing more refined articulation [35]. The primary of these muscles is the sacrocaudalis dorsalis (medialis and lateralis), sacrocaudalis ventralis (medialis and lateralis), and intertraversarii caudae. A prior study of these muscles found that they extend to different points in the tail, with *Serratus ventralis* medialis absent by the fourth joint level and *Serratus dorsalis* medialis absent by the seventh. Additionally, cross‐sectional areas of these muscles decreased distally at different rates, and the *S. dorsalis* lateralis has been implicated in contributing to both lateral bending and dorsal extension motions. These prior findings suggest both level‐wise variations in motion and potential coupling between flexion‐extension and lateral bending. As the bovine tail lacks interspinous and prominent longitudinal ligaments, along with facet joints, the IVDs present the only limits on the range of joint motion.

The bovine tail thus presents a unique opportunity to study both the patterns of dynamic motion in a large animal tail and the impact of repetitive motion on intervertebral joint range of motion resulting from multi‐scale tissue development and remodeling. This study hypothesized that motion patterns in bovine caudal motion segments depend on anatomical level. It was further hypothesized that disc levels with greatest exposure to repetitive motion would exhibit reduced range of motion subsequent to AF tissue adaptation. These hypotheses were tested using in vivo motion tracking of bovine tails along with ex vivo passive range of motion testing and geometric and microstructural measurement.

## 2 Methods

### 2.1 In Vivo Active ROM

In vivo active range of motion was measured by placing a custom designed motion tracker onto the tails of six skeletally mature, non‐lactating Holstein cows. The study was approved by the Clarkson University Institutional Animal Care and Use Committee (protocol # 22‐01) and performed in accordance with relevant guidelines and regulations. The tracker consisted of three pods containing accelerometer‐gyroscope‐magnetometer sensors (LSM6DSOX + LIS3MDL, Adafruit Industries, New York, NY) connected in series by a cable. One of the pods additionally contained a battery, microprocessor with micro SD card writer (Feather M0 Addalogger, Adafruit) and multiplexer (TCA9548A I2C, Texas Instruments, Dallas, TX).

**FIGURE 2:**
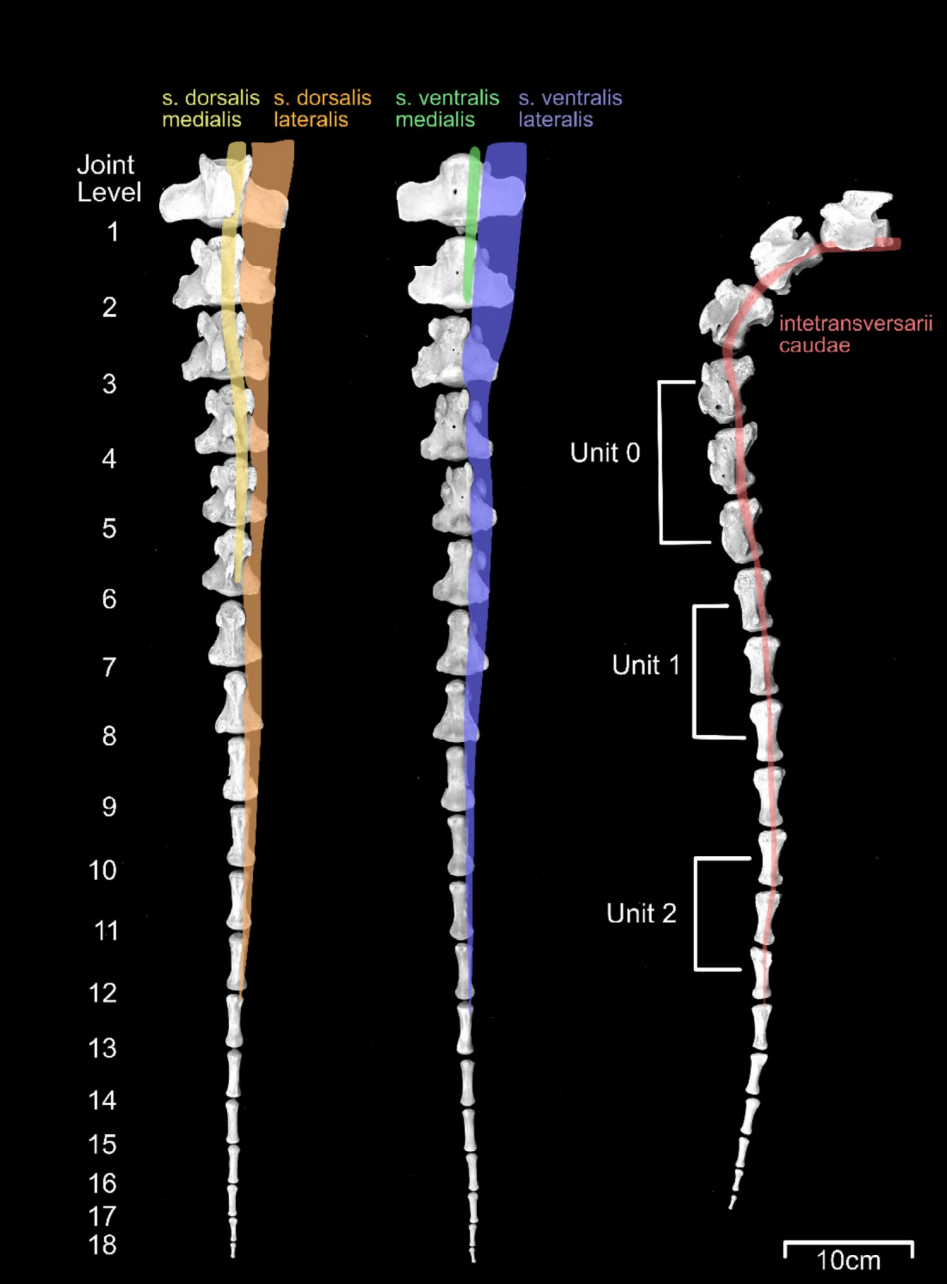
Vertebrae of the bovine tail viewed ventral (left) and dorsal (center) aspects in the fully extended posture and lateral aspect (right) with locations and extents of intrinsic muscles (as reported by Young and Kenrick [35]) shaded and ranges of in vivo sensor pods indicated.

The three pods were attached to the dorsal aspect of the tail using veterinary bandaging tape (Vetrap, 3M, St. Paul, MN). The pod with the microprocessor (Unit 0, weight 45 g) was attached approximately 15 cm from the base of the tail, while the other two (Unit 1 and Unit 2, weight 8 g each) were placed at approximately 50% and 75% of total tail length. Tail length and sensor pod placement locations were measured and recorded. Placement of Unit 0 ranged from the 4th to 6th vertebra, Unit 1 from 7th to 10th, and Unit 2 11th through 13th (Figure 2). As the total mass of an adult bovine tail is approximately 1.5 kg [34], and the heaviest pod was placed proximally, the total added mass of the tracker was not expected to significantly alter normal tail motion.

All three pods were placed with positive local *x*, *y*, and *z* axes corresponding to the animal left side, distal, and dorsal respectively. During testing, acceleration, angular velocity, and magnetic field intensity were recorded in all three directions from all three sensors at a rate of 70 Hz for 15 min. Motion tracking was performed in a barn setting at approximately 20°C. The subjects were under direct observation during tracking and exhibited no signs of distress or behavior differing from that prior to sensor placement or after sensor removal.

Motion tracker data was processed using a series of custom written Matlab codes (Mathworks Inc., Natick, MA). Joint level flexion‐extension and lateral bending angles were calculated at each time point from the tracker magnetometer readings as follows. First, magnetic field vectors were unitized and rotated into a global coordinate system such that the mid‐sagittal plane of the animal was in the global *x*–*y* plane with positive *y* vertical and positive *x* caudal. A rigid body model of the tail was constructed with segmental lengths estimated by using prior measurement [34] of bovine caudal vertebral bodies and discs scaled to the measured tail length of each subject. A cubic spline function with C^2^ continuity was used to interpolate the tracker orientation vectors, along with a < 1,0,0 > constraint at the base of the tail, to the cumulative segmental length corresponding to each vertebral body center. Flexion‐extension and lateral bending angles were then applied to successive joints in the model in order to match the segments to the interpolated orientation vectors. To avoid extrapolation errors, only the first 12 joints were considered.

Periods of time when the tail was stationary were identified by summing the absolute values of the local *x*‐ and *z*‐axis angular velocities of the three trackers, applying a median filter with a 2.8 s window, and thresholding at a total value of 1°/s. Tracker magnetometer orientations during these rest periods were used to fine‐tune the rotation to global coordinates. This was done to account for shifting of the animal over the course of data collection. Joint angles from time points identified as moving were extracted, and relative lateral bend angles of the third and eighth joints were used to segment data into modes of motion, with both angles having the same sign defining Mode 1 and opposite signs defining Mode 2.

### 2.2 Ex Vivo Passive Range of Motion

Five adult bovine tails were obtained from a local abattoir and were stored at −20°C until testing. On the day of testing, the tails were slowly warmed to room temperature, then muscles and tendons were removed. The tails were placed on the laboratory bench and manually pulled into maximum lateral bending, flexion, and extension while being photographed from above using a digital camera (Olympus E‐M5III, OM Digital Solutions, Tokyo) with a 100 mm lens. As the tails were able to bend laterally beyond a closed circle, they were photographed twice (once with proximal on top and once with distal on top) in order to see all levels of the tail. The images were then loaded into a custom written Matlab script, and the perimeters of each IVD were manually selected. The centroids of each IVD were calculated, and the difference in vectors joining adjacent pairs of IVD centroids was used to calculate joint angle. This process was repeated three times for each image. The angles of mid‐tail joints (4–8) in lateral bending were compared between photographs to test the repeatability of manual positioning.

### 2.3 Disc Geometric and Tissue Characterization

After the tails were photographed, anterior–posterior and lateral widths of the discs were measured with a dial caliper. The discs were then dissected out with a scalpel and heights were measured. Ratios of height to lateral width, height to anterior–posterior width, and lateral width to anterior–posterior width were calculated from the recorded measurements. The discs were then sectioned with a cryostat to produce 30 μm thick sections at approximately mid‐height, which were placed on glass slides (VWR, Radnor, PA), air dried, and mounted with Polyglass coverslipping medium (Polysciences, Warrington, PA). Transected discs were then thawed and photographed using a digital camera equipped with a 100 mm lens. The photographs were manually digitized using ImageJ to measure total AF thickness in the anterior, posterior, and lateral regions, along with disc width in the anterior–posterior and lateral directions. AF thickness was then normalized to the relevant width: anterior and posterior to a–p width and lateral to lateral width. This process was repeated three times for each image.

Outer AF crimp period was measured by imaging the mounted slides using an inverted microscope (Olympus IMT‐2, Olympus, Waltham, MA) equipped with crossed polarizing filters and a digital camera. Micrographs were analyzed using a Matlab script which performed a Fast Fourier Transform on three user‐selected lines of interest to yield an average crimp period [36]. To maintain consistency with the in vivo measurements detailed above, only the first 12 joint levels were considered.

### 2.4 Statistics

For in vivo active ranges of motion, the 95th percentile predicted lateral bending angles and angular velocities were calculated for each subject at each joint level, along with the difference between 95th percentile and 5th percentile flexion‐extension angle. Two‐way ANOVAs were then performed to test the effects of mode and joint level on each dependent variable. For ex vivo passive ranges of motion, a two‐way ANOVA was used to test the effects of level and direction along with interaction. Additionally, a two‐way ANOVA was used to test the effects of level and direction on total range of motion. Total range of motion was defined as the full angular span from maximum positive to maximum negative rotation about each axis. This is equal to twice the measured range in lateral bending and the sum of flexion and extension ranges in flexion‐extension. A one‐way ANOVA was used to test the effect of level on disc geometry. In all cases, a Tukey–Kramer post hoc test was applied to relevant pairwise comparisons with a significance value of *p* < 0.01. Tables of level‐wise significance tests are provided as a supplement.

## 3 Results

### 3.1 In Vivo Active Range of Motion Varies by Tail Level

**FIGURE 3:**
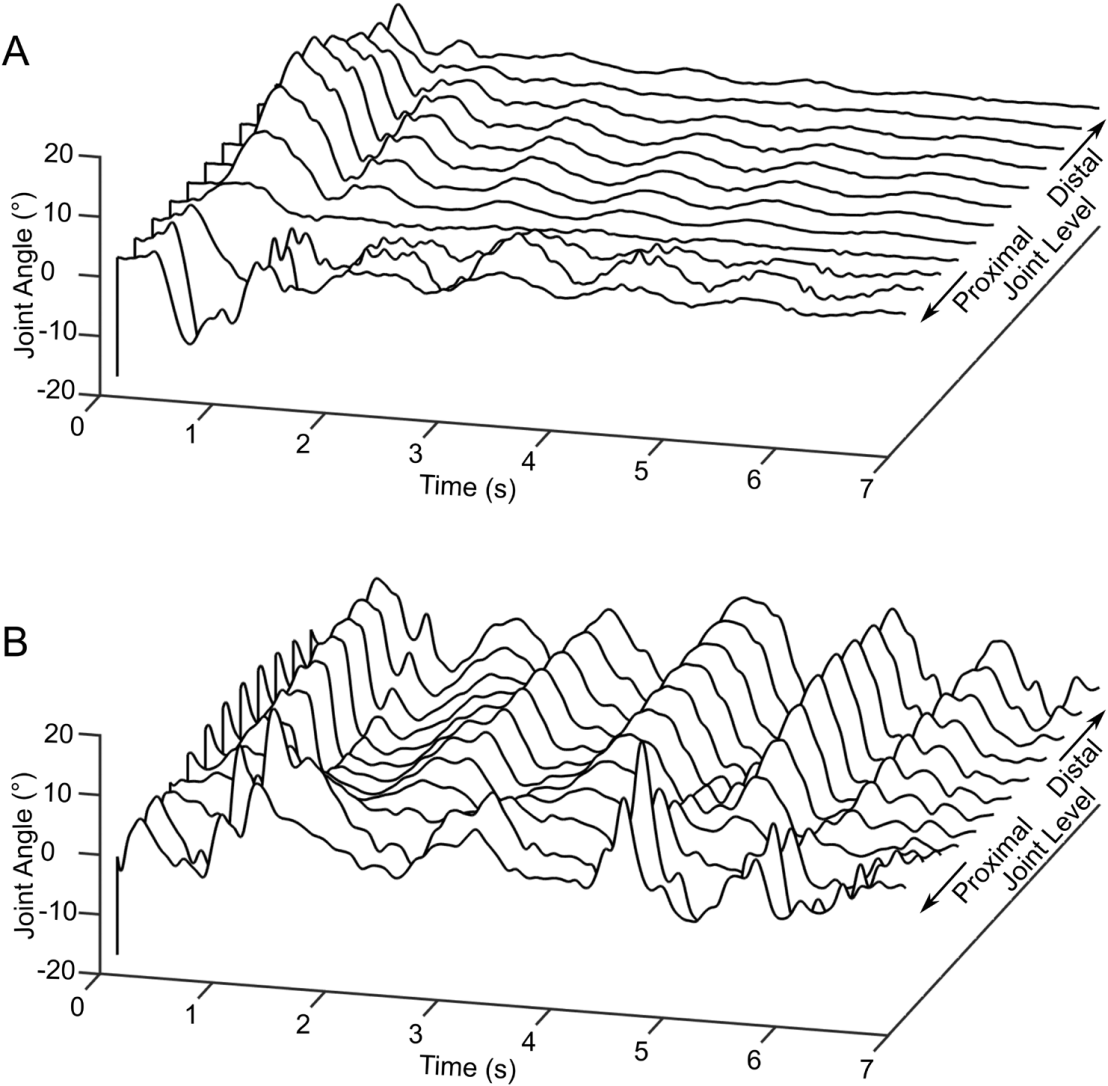
Calculated lateral bend angles for representative swishing (A) and flailing (B) type motions.

Tracking of the six live subjects yielded a total of 84 min of data comprised of 45% stationary and 55% moving time. Representative joint angle versus time data is shown in Figure 3. Data included a range of different tail motions, primarily swishing events (Figure 3A) consisting of a large impulse followed by periodic swinging with a gradually decaying amplitude and continuous, high‐amplitude flailing type behavior (Figure 3B). Of the moving time, 40% was identified as Mode 1 (lateral bending of the tail with continuous curvature, as shown in Figure 1A), while 60% of all samples were identified as Mode 2 (lateral bending with proximal and distal portions of the tail curved in opposite directions, as shown in Figure 1B).

**FIGURE 4:**
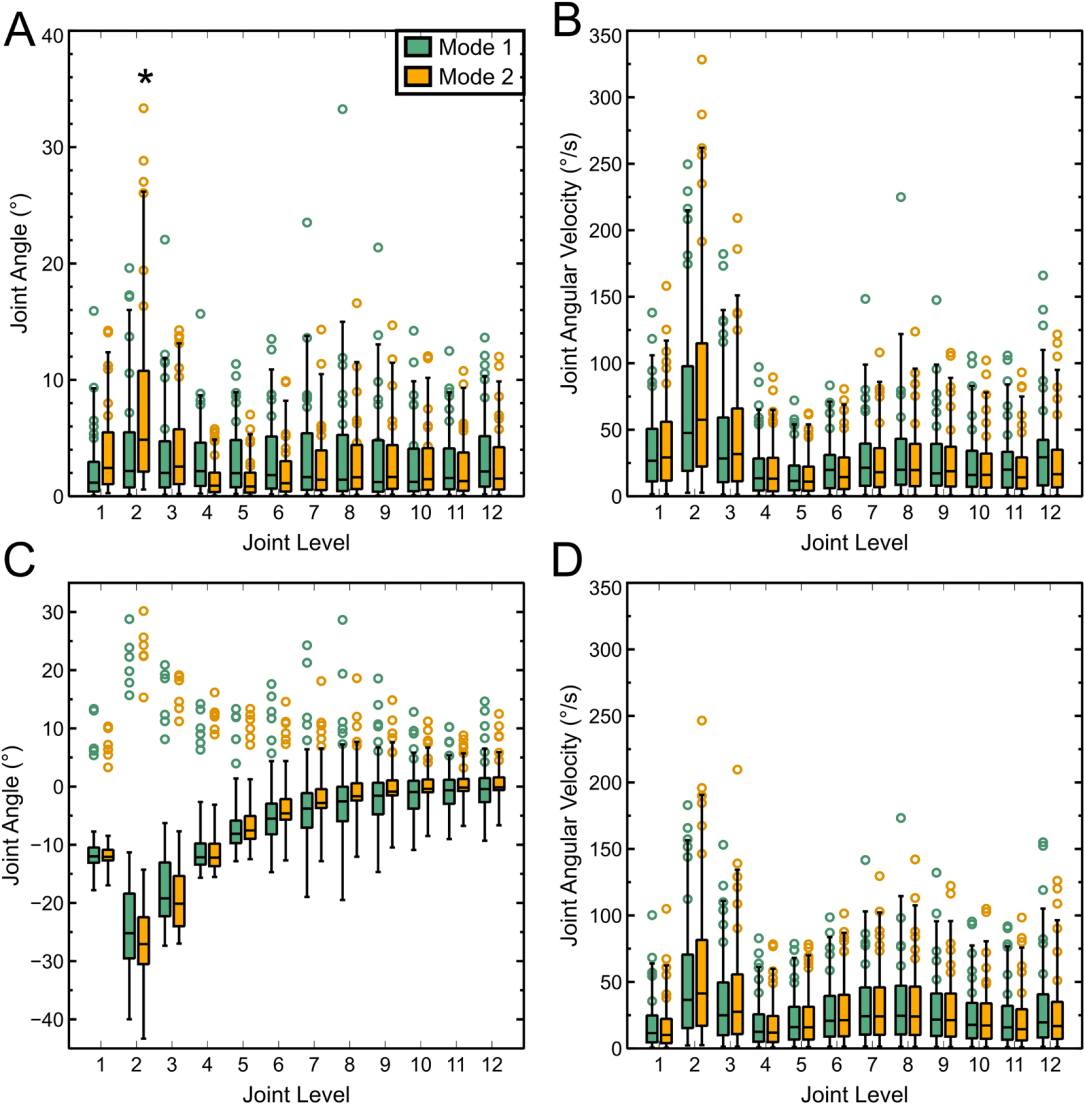
Median, Quartile, and 95% range of all calculated lateral bending angles (A), absolute lateral bend angular velocities (B), flexion‐extension angles (C), and absolute flexion‐extension angular velocities (D) during Mode 1 and Mode 2 motion for six animal subjects. Negative angles in C indicate forward flexion, and positive angles indicate extension. Circles indicate 95th percentile values for each of the subjects in A, B, and D and differences between 95th and 5th percentiles in C. * indicates a significant difference (*p* < 0.01) between modes.

Figure 4 presents lateral bend and flexion‐extension angles and angular velocities for all moving time, segmented by mode shape. Raw angles and velocities are plotted as medians, quartiles, and ranges in order to visualize how much time each joint is spending at each angle. Circles in Figure 4 indicate the 95th percentile for each individual animal subject, which was used to make the following statistical comparisons. In lateral bending (Figure 4A), there was a significant effect of level (*p* < 0.001), but not mode (*p* = 0.113) on 95th percentile lateral bend angle. Median absolute joint angles in Mode 1 were relatively constant with joint level; however, the 95th percentile range had peaks at the second and eighth joints and a minimum at the fourth joint. In Mode 2, there was a large peak in both median and range at the second joint and a more pronounced minimum in both median and range between the fourth and fifth joints, consistent with the location of the inflection point seen in Figure 1A. The 95th percentile of lateral bending angular velocity (Figure 3B) was similarly affected by level (*p* < 0.001), but not mode (*p* = 0.75). In flexion‐extension, motion occurred largely about the resting tail posture (Figure 4C). The difference between 5th and 95th percentile angles varied significantly with level (*p* < 0.001), but not mode (*p* = 0.21). This range peaked at the second and eighth joints with a minimum at the fourth. Flexion‐extension angular velocity (Figure 4D) was generally lower in magnitude than lateral bend velocity and varied significantly with level (*p* < 0.001) but not mode (*p* = 0.22).

### 3.2 Ex Vivo Passive Range of Motion Varies by Level and Axis

**FIGURE 5:**
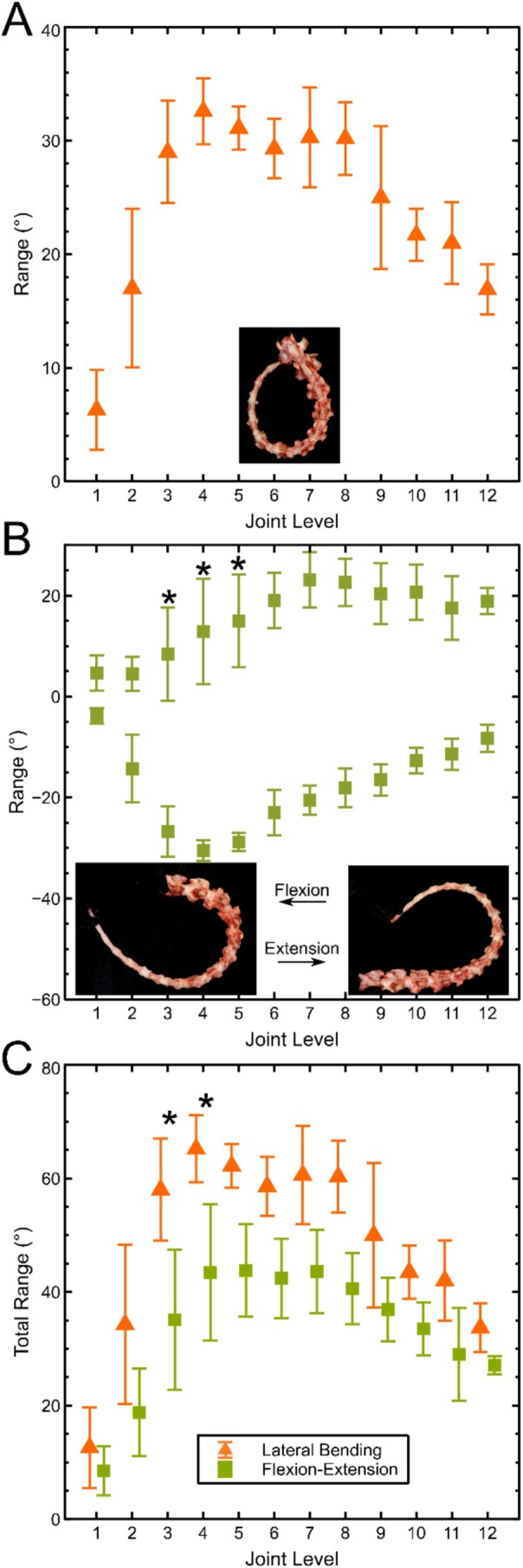
Passive ex vivo ranges of motion relative to a straight tail by joint level in lateral bending (A), flexion and extension (B, where negative values are flexion, and positive are extension). Total ranges of motion (C) for five tails. * indicates a significant (*p* < 0.01) difference between the absolute flexion range and extension range in B and between the total lateral bending range and total flexion‐extension range in C. Inserts in A and B show typical images used for analysis.

Average passive joint range of motion across all five tails varied significantly by both level (*p* < 0.001) and axis (*p* < 0.001) as shown in Figure 5. In lateral bending, the tail formed a teardrop shape with a minimum radius around the sixth joint level (Figure 5A, inset). Average lateral bend angles (Figure 5A) were lowest in proximal joints, rising to a broad peak across the third through eighth joints, before decreasing again in the distal tail. Flexion and extension angles (Figure 5B) were similarly lowest in proximal joints and varied significantly along the length of the tail, but while the forward flexion angle peaked in the fourth joint, the extension angle peaked in the seventh. Total range of motion, which represents the angle from maximum positive to maximum negative rotation, was defined as twice the lateral bend angle in lateral bending, and the sum of absolute flexion and extension angles in flexion‐extension showed similar level‐wise trends (Figure 5C) but was consistently higher in lateral bending. Repeatability of the manual range of motion measurement was assessed by calculating joint level 4–8 lateral bend angle from two photographs of each tail, yielding an average error of 1.8°. The standard deviation across tails at these levels averaged 3.5°.

### 3.3 IVD Geometry, Mesostructure, and Microstructure Vary by Level

**FIGURE 6:**
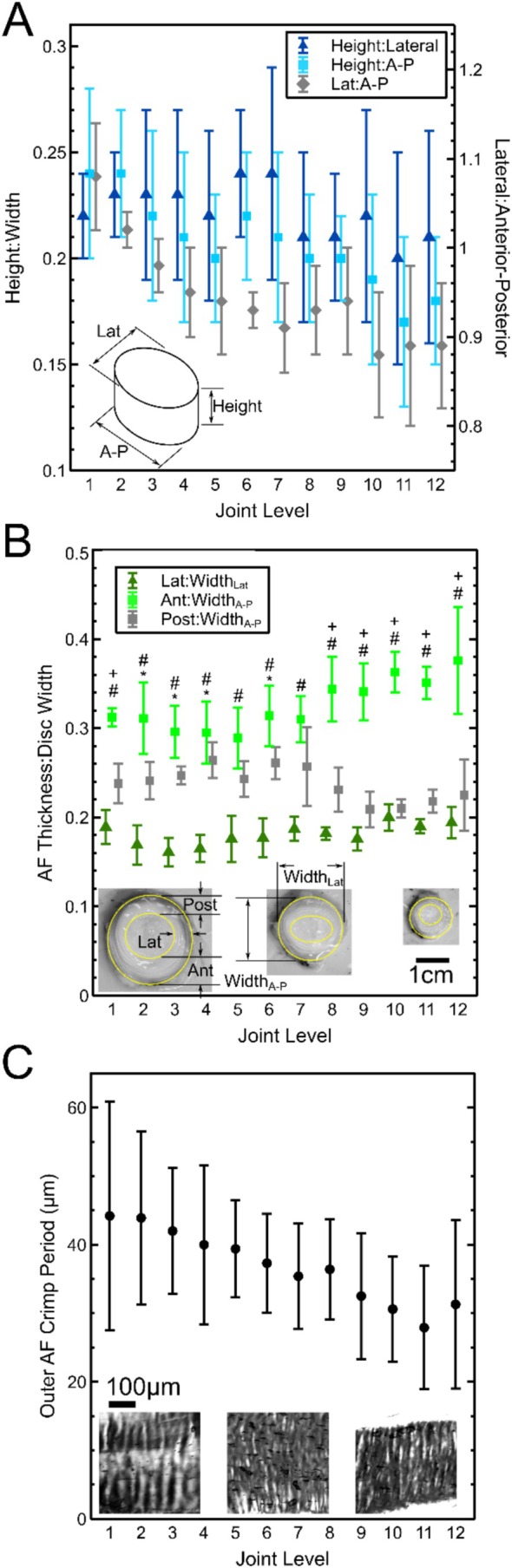
Average ± SD values for the ratio of disc height to width in lateral and anterior–posterior (A–P) directions, along with the ratio of lateral to anterior–posterior width (A), total AF radial thickness normalized to disc width (B), and outer AF fiber crimp period (C) for five tails. Insets in B and C are representative images from the first, sixth, and twelfth discs. In B, + indicates the difference between Anterior and Posterior, # indicates the difference between Anterior and Lateral, and * indicates the difference between posterior and lateral (all *p* < 0.01).

Disc aspect ratios varied nonsignificantly with level (*p* = 0.064) and direction (*p* = 0.06), with height decreasing relative to both lateral width and anterior–posterior width when moving from proximal to distal tail. In the transverse plane, there was a significant effect (*p* < 0.0001) of level on anterior–posterior ratio (Figure 6A), decreasing from 1.08 in the proximal tail to 0.89 distally. Though not significant, height relative to lateral width became increasingly greater than height relative to anterior–posterior width moving distally.

Annulus fibrosus radial thickness normalized to disc width (Figure 6B) varied by both level (*p* = 0.0497) and region (*p* < 0.0001) with a significant interaction (*p* < 0.0001). At all levels, normalized AF thickness was highest in the anterior region, followed by posterior, then lateral. In levels 8–12, normalized anterior thickness further increased, and normalized posterior thickness further decreased. There was no significant effect of region on outer AF crimp period. Pooled crimp period around the whole AF (Figure 6C) had a significant (*p* < 0.0001) monotonic decrease along the tail.

## 4 Discussion

The results of this study support the hypothesis that joint levels in the bovine tail experience different amounts of repetitive flexion‐extension and lateral bending in vivo. Despite being nearly cylindrical and having similar height to width ratios, the range of bending motion of the bovine IVD similarly varies significantly by both level and by bending axis. With the exception of the first joint, the levels exposed to the lowest amounts of bending motion in vivo were found to have the highest passive range of motion. This effect is not readily explained by any single trend in disc geometry, AF radial thickness, AF fiber crimping, or disc composition.

The observed in vivo motion of the bovine tail generally agrees with the prior work by Matherne et al. [32] which suggested that animal tails are typically not driven at their natural frequency. Tail motion in the present study had an average period of approximately 1 Hz (Figure 2), which Matherne et al. would predict for an animal of this size. For a classical double pendulum in stable oscillation, the natural frequency of Mode 2 is higher than that of Mode 1 [37]. However, the comparable ranges in angular amplitude and angular velocity shown in Figure 3 suggest that this is not the case in the tail.

As expected, the median values of lateral bend angle (Figure 4) are relatively constant along the tail while it is in Mode 1. This is consistent with the constant radius assumption presented by Duclos et al. [34] However, the 95% ranges peak at the same joint levels that they do in Mode 2. Furthermore, minima are seen in the variance of flexion‐extension in both modes at the same level as the Mode 2 lateral bending inflection point. This suggests that the muscle loading patterns associated with Mode 2 lateral bending also affect how the tail actively moves in Mode 1 bending. The observation of this inflection point in flexion‐extension is consistent with the *S. dorsalis* lateralis being active in both extension and lateral bending movements [35].

The present study confirms a lack of correlation between aspect ratio and range of motion in the IVD. A prior study of IVD geometry [11] reported a height to width ratio in the human IVD of approximately 0.3 in the anterior–posterior direction and 0.2 in the lateral direction. According to elastic beam theory, rotation of a homogenous prism under an applied moment is proportional to length (in this case height) divided by second moment of area. This would suggest greater flexibility when the moment is applied about the major axis of the disc's transverse cross‐section (flexion‐extension) than about the minor axis (lateral bending). However, the ranges of motion in healthy human lumbar discs have been reported [38] to be 10° in flexion‐extension and 16° in lateral bending. As in the present study, these ranges are contrary to the greater exposure to flexion‐extension than lateral bending during human activities of daily living [39]. Similarly, the height to width ratio of bovine caudal discs is comparable in both directions, yet the total passive range of motion varies from approximately 10°–40° in flexion extension and approximately 15°–65° in lateral bending. While our measurements showed that the height to width ratio was slightly higher in the lateral direction than anterior–posterior, the difference was largest where the difference in range of motion was smallest (at more distal levels). The contradiction between the lack of level‐wise trends in composition and geometry in bovine caudal IVDs and the presently observed trend in passive range of motion suggests that the mechanical behavior of the disc is highly sensitive to small changes at the tissue level.

**FIGURE 7:**
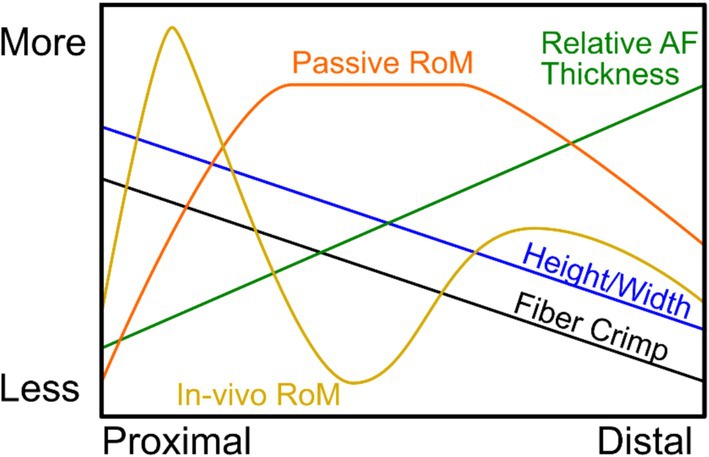
Schematic overview of level‐wise trends.

A summary of level‐wise trends found in this study is presented schematically in Figure 7. Tissue level adaptation to motion was investigated through AF radial thickness (presumed to be established during development; [2]) and AF fiber crimp (presumed to result from microscale remodeling within the mature‐state disc [9, 34]). A decreasing trend in outer AF fiber crimp period has been previously reported from bovine tail IVDs from the first through fifth joints [34]. Crimp period is associated with nonlinear tissue behavior [40], with the transition from fiber uncrimping to fiber stretching resulting in an increase in stiffness. Assuming comparable fiber composition and structure, a higher initial crimp period indicates that fibers are prestretched and may undergo a smaller amount of additional tensile strain before stiffening. The previously described level‐wise decrease is thus supported by the observed increase in lateral bending range of motion across these levels. However, in the present study, outer AF fiber crimp period was found to continue decreasing monotonically through the twelfth joint level despite passive ranges of motion also decreasing. Similarly, increasing AF thickness on the convex and concave sides of a flexed disc would be expected to reduce range of motion. This is broadly supported by the lateral AF having smaller relative AF thickness than anterior and posterior and a higher measured range of motion in lateral bending than flexion‐extension. Additionally, lateral AF relative thickness is slightly lower in the mid‐tail joints where range of motion is highest. However, while there is a similar level‐wise relationship between anterior AF relative thickness and range of motion in extension, there is not between posterior AF thickness and flexion. In the proximal bovine tail, the posterior AF was previously shown to have a different distribution of circumferential residual strain (with tension in both inner and outer AF and compression in the middle) than anterior and lateral (trending monotonically from compression to tension, inner to outer). Taken together, these findings suggest that adaptations in different joint levels occur at different time points, warranting further study of immature subjects.

While the bovine tail IVD is a frequently used experimental model, neither in vivo nor ex vivo joint ranges of motion have previously been reported. As a result, prior mechanical studies using this species have based lateral bending and flexion‐extension amplitudes on the human lumbar range of motion [22]. Interestingly, the presently measured active and passive ranges of motion are higher in both directions, and at most levels, than the 15° previously shown to induce degenerative changes when applied statically to the bovine disc [5]. The results of this study confirm that extreme care should be taken when using bovine caudal IVDs as a research model. Despite their cylindrical outside geometry, they may not be assumed to be axisymmetric mechanically, nor may anatomical levels be treated interchangeably, and the bovine disc's range of motion may not be assumed to equal that of a human's.

While the in vivo motion tracking data used in this study contains a sufficient number and type of motion events to fully characterize the ranges of motion required to perform them, it represents a small amount of collection time and may not necessarily extrapolate to the number of motion events in a day, week, month, or year. It was noted that the measured subjects displayed a wide range of activity levels during data collection. For example, the outlying values between the 7th and 9th joints seen in Figure 3 are attributed to a subject whose tail was in high amplitude flailing motion during most of the time of data collection. Additionally, in vivo joint angles were calculated by interpolating between orientations of three trackers and a stationary tail root rather than directly. Placements of the three sensors of the tracker ranged from the 4rd through 6th joint for the first sensor, the 7th through 10th for the second, and the 11th through 13th for the third. The location of the Mode 2 inflection point thus occurred proximally or distally to the first sensor in individual subjects, suggesting that it was not an artifact of interpolation.

The findings of this study have important implications beyond the movement of animal tails. Passive stiffness of human lumbar IVDs is a key contributor to spinal column stability [41]. Repetitive flexion‐extension motion has been shown to acutely increase lumbar laxity through viscoelastic mechanisms [42]. Over time, repetitive flexion is associated with a decrease in lumbar range of motion [43]. The bovine tail may provide the ideal model system for probing the timeline and underlying mechanisms of this process without the cost and ethical concerns of using laboratory‐bred animal subjects. It should be noted that the in vivo motion tracking in this study was performed between June and August, and ex vivo testing was performed using tails obtained in June. Given the swatting function of the bovine tail and the seasonal variability in fly population, testing throughout the rest of the year is an important avenue of future research. In particular, charting both in vivo motion exposure and ex vivo range of motion throughout the year may establish the rate at which tissue adaptation occurs in a large mammalian IVD, informing injury mitigation strategies for humans who are subjected to changes in activity level, such as change of occupation, participation in seasonal sports, or recovery from injury.

In conclusion, different joint levels in the bovine tail are exposed to different levels of both flexion‐extension and lateral bending as a result of both the resting posture of the tail and active motions. As a result, passive flexural motion varies by both level and bending axis, with higher motion exposure associated with lower passive range of motion. These trends are not readily explained by any single macro‐ (disc aspect ratio), meso‐ (relative AF radial thickness), or micro‐ (outer AF crimp period) scale feature, suggesting complex mechanobiological processes during tail growth employing different mechanisms during growth and in the mature state. These results suggest that further study of bovine tail mechanics at different time points may offer powerful insights into how human lumbar IVDs adapt in response to repetitive motions.

## Conflicts of Interest

The authors declare no conflicts of interest.

## Supporting information


**Data S1.** Supporting Information.

## Data Availability

Raw data is available from the corresponding author upon request.
