# Supplementary material for: Complex In Vivo Motion of the Bovine Tail Provides Unique Insights Into Intervertebral Disc Adaptation
Source: JOR Spine. 2025 Jun 17;8(2):e70084. doi: 10.1002/jsp2.70084 (PMC12172803; doi:10.1002/jsp2.70084)
Supplement: Supplementary file 1 — Data S1. Supporting Information. [file JSP2-8-e70084-s001.doc]

# Supplemental Material: Level-Wise Post-Hoc Comparisons

For in-vivo active ranges of motion, the 95^th^ percentile predicted lateral bending angles and angular velocities were calculated for each subject at each joint level, along with the difference between 95^th^ percentile and 5^th^ percentile flexion-extension angle. Two-way ANOVAs were then performed to test the effects of mode and joint level on each dependent variable. For ex-vivo passive ranges of motion, a two-way ANOVA was used to test the effects of level and direction along with interaction. Additionally, a two-way ANOVA was used to test the effects of level and direction on total range of motion. A one-way ANOVA was used to test the effect of level on disc geometry. In all cases, a Tukey-Kramer post-hoc test was applied to relevant pairwise comparisons with a significance value of p<0.01. The grids below contain the results of the Tukey-Kramer post-hoc test between pairs of joint levels (For example, Row 1, Column 3 compares level c1-c2 with level c3-c4) for each dependent variable, with X indicating p<0.01.

## In-Vivo Range of Motion, Pooled by Subject

### Lateral Bending (95^th^ percentile)

#### Mode 1

|  | 2 | 3 | 4 | 5 | 6 | 7 | 8 | 9 | 10 | 11 | 12 |
| --- | --- | --- | --- | --- | --- | --- | --- | --- | --- | --- | --- |
| 1 | O | O | O | O | O | O | O | O | O | O | O |
| 2 |  | O | O | O | O | O | O | O | O | O | O |
| 3 |  |  | O | O | O | O | O | O | O | O | O |
| 4 |  |  |  | O | O | O | O | O | O | O | O |
| 5 |  |  |  |  | O | O | O | O | O | O | O |
| 6 |  |  |  |  |  | O | O | O | O | O | O |
| 7 |  |  |  |  |  |  | O | O | O | O | O |
| 8 |  |  |  |  |  |  |  | O | O | O | O |
| 9 |  |  |  |  |  |  |  |  | O | O | O |
| 10 |  |  |  |  |  |  |  |  |  | O | O |
| 11 |  |  |  |  |  |  |  |  |  |  | O |

#### Mode 2

|  | 2 | 3 | 4 | 5 | 6 | 7 | 8 | 9 | 10 | 11 | 12 |
| --- | --- | --- | --- | --- | --- | --- | --- | --- | --- | --- | --- |
| 1 | O | O | O | O | O | O | O | O | O | O | O |
| 2 |  | O | O | O | O | O | O | O | O | O | **X** |
| 3 |  |  | O | O | O | O | O | O | O | O | O |
| 4 |  |  |  | O | O | O | O | O | O | O | O |
| 5 |  |  |  |  | O | O | O | O | O | O | O |
| 6 |  |  |  |  |  | O | O | O | O | O | O |
| 7 |  |  |  |  |  |  | O | O | O | O | O |
| 8 |  |  |  |  |  |  |  | O | O | O | O |
| 9 |  |  |  |  |  |  |  |  | O | O | O |
| 10 |  |  |  |  |  |  |  |  |  | O | O |
| 11 |  |  |  |  |  |  |  |  |  |  | O |

### Lateral Bend Angular Velocity (95^th^ percentile)

#### Mode 1

|  | 2 | 3 | 4 | 5 | 6 | 7 | 8 | 9 | 10 | 11 | 12 |
| --- | --- | --- | --- | --- | --- | --- | --- | --- | --- | --- | --- |
| 1 | **X** | O | O | O | O | O | O | O | O | O | O |
| 2 |  | O | **X** | **X** | **X** | **X** | **X** | **X** | **X** | **X** | **X** |
| 3 |  |  | **X** | **X** | **X** | O | O | O | **X** | O | O |
| 4 |  |  |  | O | O | O | O | O | O | O | O |
| 5 |  |  |  |  | O | O | O | O | O | O | O |
| 6 |  |  |  |  |  | O | O | O | O | O | O |
| 7 |  |  |  |  |  |  | O | O | O | O | O |
| 8 |  |  |  |  |  |  |  | O | O | O | O |
| 9 |  |  |  |  |  |  |  |  | O | O | O |
| 10 |  |  |  |  |  |  |  |  |  | O | O |
| 11 |  |  |  |  |  |  |  |  |  |  | O |

#### Mode 2

|  | 2 | 3 | 4 | 5 | 6 | 7 | 8 | 9 | 10 | 11 | 12 |
| --- | --- | --- | --- | --- | --- | --- | --- | --- | --- | --- | --- |
| 1 | **X** | O | O | O | O | O | O | O | O | O | O |
| 2 |  | **X** | **X** | **X** | **X** | **X** | **X** | **X** | **X** | **X** | **X** |
| 3 |  |  | **X** | **X** | **X** | **X** | **X** | **X** | **X** | **X** | O |
| 4 |  |  |  | O | O | O | O | O | O | O | O |
| 5 |  |  |  |  | O | O | O | O | O | O | O |
| 6 |  |  |  |  |  | O | O | O | O | O | O |
| 7 |  |  |  |  |  |  | O | O | O | O | O |
| 8 |  |  |  |  |  |  |  | O | O | O | O |
| 9 |  |  |  |  |  |  |  |  | O | O | O |
| 10 |  |  |  |  |  |  |  |  |  | O | O |
| 11 |  |  |  |  |  |  |  |  |  |  | O |

### Flexion Extension (95^th^ percentile minus 5^th^)

#### Mode 1

|  | 2 | 3 | 4 | 5 | 6 | 7 | 8 | 9 | 10 | 11 | 12 |
| --- | --- | --- | --- | --- | --- | --- | --- | --- | --- | --- | --- |
| 1 | **X** | O | **X** | X | O | O | O | **X** | **X** | **X** | **X** |
| 2 |  | O | O | O | O | O | O | O | O | O | O |
| 3 |  |  | O | O | O | O | O | O | O | O | O |
| 4 |  |  |  | O | O | O | O | O | O | O | O |
| 5 |  |  |  |  | O | O | O | O | O | O | O |
| 6 |  |  |  |  |  | O | O | O | O | O | O |
| 7 |  |  |  |  |  |  | O | O | O | O | O |
| 8 |  |  |  |  |  |  |  | O | O | O | O |
| 9 |  |  |  |  |  |  |  |  | O | O | O |
| 10 |  |  |  |  |  |  |  |  |  | O | O |
| 11 |  |  |  |  |  |  |  |  |  |  | O |

#### Mode 2

|  | 2 | 3 | 4 | 5 | 6 | 7 | 8 | 9 | 10 | 11 | 12 |
| --- | --- | --- | --- | --- | --- | --- | --- | --- | --- | --- | --- |
| 1 | **X** | O | O | O | O | O | O | O | O | O | O |
| 2 |  | O | **X** | **X** | **X** | **X** | **X** | **X** | **X** | **X** | **X** |
| 3 |  |  | O | O | O | O | O | O | O | O | O |
| 4 |  |  |  | O | O | O | O | O | O | O | O |
| 5 |  |  |  |  | O | O | O | O | O | O | O |
| 6 |  |  |  |  |  | O | O | O | O | O | O |
| 7 |  |  |  |  |  |  | O | O | O | O | O |
| 8 |  |  |  |  |  |  |  | O | O | O | O |
| 9 |  |  |  |  |  |  |  |  | O | O | O |
| 10 |  |  |  |  |  |  |  |  |  | O | O |
| 11 |  |  |  |  |  |  |  |  |  |  | O |

### Flexion Velocity (95^th^ percentile of absolute value)

#### Mode 1

|  | 2 | 3 | 4 | 5 | 6 | 7 | 8 | 9 | 10 | 11 | 12 |
| --- | --- | --- | --- | --- | --- | --- | --- | --- | --- | --- | --- |
| 1 | **X** | O | O | O | O | O | O | O | O | O | O |
| 2 |  | O | **X** | **X** | **X** | **X** | O | **X** | **X** | **X** | O |
| 3 |  |  | O | O | O | O | O | O | O | O | O |
| 4 |  |  |  | O | O | O | O | O | O | O | O |
| 5 |  |  |  |  | O | O | O | O | O | O | O |
| 6 |  |  |  |  |  | O | O | O | O | O | O |
| 7 |  |  |  |  |  |  | O | O | O | O | O |
| 8 |  |  |  |  |  |  |  | O | O | O | O |
| 9 |  |  |  |  |  |  |  |  | O | O | O |
| 10 |  |  |  |  |  |  |  |  |  | O | O |
| 11 |  |  |  |  |  |  |  |  |  |  | O |

#### Mode 2

|  | 2 | 3 | 4 | 5 | 6 | 7 | 8 | 9 | 10 | 11 | 12 |
| --- | --- | --- | --- | --- | --- | --- | --- | --- | --- | --- | --- |
| 1 | **X** | **X** | O | O | O | O | O | O | O | O | O |
| 2 |  | O | **X** | **X** | **X** | **X** | **X** | **X** | **X** | **X** | **X** |
| 3 |  |  | **X** | **X** | O | O | O | O | O | **X** | O |
| 4 |  |  |  | O | O | O | O | O | O | O | O |
| 5 |  |  |  |  | O | O | O | O | O | O | O |
| 6 |  |  |  |  |  | O | O | O | O | O | O |
| 7 |  |  |  |  |  |  | O | O | O | O | O |
| 8 |  |  |  |  |  |  |  | O | O | O | O |
| 9 |  |  |  |  |  |  |  |  | O | O | O |
| 10 |  |  |  |  |  |  |  |  |  | O | O |
| 11 |  |  |  |  |  |  |  |  |  |  | O |

## Ex-Vivo Range of Motion

### Flexion

|  | 2 | 3 | 4 | 5 | 6 | 7 | 8 | 9 | 10 | 11 | 12 |
| --- | --- | --- | --- | --- | --- | --- | --- | --- | --- | --- | --- |
| 1 | O | **X** | **X** | **X** | **X** | **X** | **X** | O | O | O | O |
| 2 |  | O | **X** | **X** | O | O | O | O | O | O | O |
| 3 |  |  | O | O | O | O | O | O | **X** | **X** | **X** |
| 4 |  |  |  | O | O | O | O | **X** | **X** | **X** | **X** |
| 5 |  |  |  |  | O | O | O | O | **X** | **X** | **X** |
| 6 |  |  |  |  |  | O | O | O | O | O | **X** |
| 7 |  |  |  |  |  |  | O | O | O | O | O |
| 8 |  |  |  |  |  |  |  | O | O | O | O |
| 9 |  |  |  |  |  |  |  |  | O | O | O |
| 10 |  |  |  |  |  |  |  |  |  | O | O |
| 11 |  |  |  |  |  |  |  |  |  |  | O |

### Extension

|  | 2 | 3 | 4 | 5 | 6 | 7 | 8 | 9 | 10 | 11 | 12 |
| --- | --- | --- | --- | --- | --- | --- | --- | --- | --- | --- | --- |
| 1 | O | O | O | O | **X** | **X** | **X** | **X** | **X** | **X** | **X** |
| 2 |  | O | O | O | **X** | **X** | **X** | **X** | **X** | O | **X** |
| 3 |  |  | O | O | O | **X** | **X** | O | O | O | O |
| 4 |  |  |  | O | O | O | O | O | O | O | O |
| 5 |  |  |  |  | O | O | O | O | O | O | O |
| 6 |  |  |  |  |  | O | O | O | O | O | O |
| 7 |  |  |  |  |  |  | O | O | O | O | O |
| 8 |  |  |  |  |  |  |  | O | O | O | O |
| 9 |  |  |  |  |  |  |  |  | O | O | O |
| 10 |  |  |  |  |  |  |  |  |  | O | O |
| 11 |  |  |  |  |  |  |  |  |  |  | O |

### Lateral Bending

|  | 2 | 3 | 4 | 5 | 6 | 7 | 8 | 9 | 10 | 11 | 12 |
| --- | --- | --- | --- | --- | --- | --- | --- | --- | --- | --- | --- |
| 1 | O | **X** | **X** | **X** | **X** | **X** | **X** | **X** | **X** | **X** | O |
| 2 |  | O | **X** | **X** | O | **X** | O | O | O | O | O |
| 3 |  |  | O | O | O | O | O | O | O | O | O |
| 4 |  |  |  | O | O | O | O | O | O | O | **X** |
| 5 |  |  |  |  | O | O | O | O | O | O | O |
| 6 |  |  |  |  |  | O | O | O | O | O | O |
| 7 |  |  |  |  |  |  | O | O | O | O | **X** |
| 8 |  |  |  |  |  |  |  | O | O | O | **X** |
| 9 |  |  |  |  |  |  |  |  | O | O | O |
| 10 |  |  |  |  |  |  |  |  |  | O | O |
| 11 |  |  |  |  |  |  |  |  |  |  | O |

## Total Range of Motion

### Flexion-Extension

|  | 2 | 3 | 4 | 5 | 6 | 7 | 8 | 9 | 10 | 11 | 12 |
| --- | --- | --- | --- | --- | --- | --- | --- | --- | --- | --- | --- |
| 1 | O | **X** | **X** | **X** | **X** | **X** | **X** | **X** | **X** | O | O |
| 2 |  | O | **X** | **X** | **X** | **X** | **X** | O | O | O | O |
| 3 |  |  | O | O | O | O | O | O | O | O | O |
| 4 |  |  |  | O | O | O | O | O | O | O | O |
| 5 |  |  |  |  | O | O | O | O | O | O | O |
| 6 |  |  |  |  |  | O | O | O | O | O | O |
| 7 |  |  |  |  |  |  | O | O | O | O | O |
| 8 |  |  |  |  |  |  |  | O | O | O | O |
| 9 |  |  |  |  |  |  |  |  | O | O | O |
| 10 |  |  |  |  |  |  |  |  |  | O | O |
| 11 |  |  |  |  |  |  |  |  |  |  | O |

### Lateral Bending

|  | 2 | 3 | 4 | 5 | 6 | 7 | 8 | 9 | 10 | 11 | 12 |
| --- | --- | --- | --- | --- | --- | --- | --- | --- | --- | --- | --- |
| 1 | **X** | **X** | **X** | **X** | **X** | **X** | **X** | **X** | **X** | **X** | **X** |
| 2 |  | **X** | **X** | **X** | **X** | **X** | **X** | O | O | O | O |
| 3 |  |  | O | O | O | O | O | O | O | O | **X** |
| 4 |  |  |  | O | O | O | O | O | **X** | **X** | **X** |
| 5 |  |  |  |  | O | O | O | O | O | O | **X** |
| 6 |  |  |  |  |  | O | O | O | O | O | **X** |
| 7 |  |  |  |  |  |  | O | O | O | O | **X** |
| 8 |  |  |  |  |  |  |  | O | O | O | **X** |
| 9 |  |  |  |  |  |  |  |  | O | O | O |
| 10 |  |  |  |  |  |  |  |  |  | O | O |
| 11 |  |  |  |  |  |  |  |  |  |  | O |

## Disc Aspect Ratio

### Height/Lateral Width

|  | 2 | 3 | 4 | 5 | 6 | 7 | 8 | 9 | 10 | 11 | 12 |
| --- | --- | --- | --- | --- | --- | --- | --- | --- | --- | --- | --- |
| 1 | O | O | O | O | O | O | O | O | O | O | O |
| 2 |  | O | O | O | O | O | O | O | O | O | O |
| 3 |  |  | O | O | O | O | O | O | O | O | O |
| 4 |  |  |  | O | O | O | O | O | O | O | O |
| 5 |  |  |  |  | O | O | O | O | O | O | O |
| 6 |  |  |  |  |  | O | O | O | O | O | O |
| 7 |  |  |  |  |  |  | O | O | O | O | O |
| 8 |  |  |  |  |  |  |  | O | O | O | O |
| 9 |  |  |  |  |  |  |  |  | O | O | O |
| 10 |  |  |  |  |  |  |  |  |  | O | O |
| 11 |  |  |  |  |  |  |  |  |  |  | O |

### Height/A-P Width

|  | 2 | 3 | 4 | 5 | 6 | 7 | 8 | 9 | 10 | 11 | 12 |
| --- | --- | --- | --- | --- | --- | --- | --- | --- | --- | --- | --- |
| 1 | O | O | O | O | O | O | O | O | O | O | O |
| 2 |  | O | O | O | O | O | O | O | O | O | O |
| 3 |  |  | O | O | O | O | O | O | O | O | O |
| 4 |  |  |  | O | O | O | O | O | O | O | O |
| 5 |  |  |  |  | O | O | O | O | O | O | O |
| 6 |  |  |  |  |  | O | O | O | O | O | O |
| 7 |  |  |  |  |  |  | O | O | O | O | O |
| 8 |  |  |  |  |  |  |  | O | O | O | O |
| 9 |  |  |  |  |  |  |  |  | O | O | O |
| 10 |  |  |  |  |  |  |  |  |  | O | O |
| 11 |  |  |  |  |  |  |  |  |  |  | O |

### Lateral Width/A-P Width

|  | 2 | 3 | 4 | 5 | 6 | 7 | 8 | 9 | 10 | 11 | 12 |
| --- | --- | --- | --- | --- | --- | --- | --- | --- | --- | --- | --- |
| 1 | O | O | O | O | **X** | **X** | O | O | **X** | **X** | **X** |
| 2 |  | O | O | O | O | O | O | O | O | O | O |
| 3 |  |  | O | O | O | O | O | O | O | O | O |
| 4 |  |  |  | O | O | O | O | O | O | O | O |
| 5 |  |  |  |  | O | O | O | O | O | O | O |
| 6 |  |  |  |  |  | O | O | O | O | O | O |
| 7 |  |  |  |  |  |  | O | O | O | O | O |
| 8 |  |  |  |  |  |  |  | O | O | O | O |
| 9 |  |  |  |  |  |  |  |  | O | O | O |
| 10 |  |  |  |  |  |  |  |  |  | O | O |
| 11 |  |  |  |  |  |  |  |  |  |  | O |

## Annulus Fibrosus Thickness

### Anterior/A-P Width

|  | 2 | 3 | 4 | 5 | 6 | 7 | 8 | 9 | 10 | 11 | 12 |
| --- | --- | --- | --- | --- | --- | --- | --- | --- | --- | --- | --- |
| 1 | O | O | O | O | O | O | O | O | O | O | O |
| 2 |  | O | O | O | O | O | O | O | O | O | O |
| 3 |  |  | O | O | O | O | O | O | O | O | **X** |
| 4 |  |  |  | O | O | O | O | O | O | O | **X** |
| 5 |  |  |  |  | O | O | O | O | **X** | O | **X** |
| 6 |  |  |  |  |  | O | O | O | O | O | O |
| 7 |  |  |  |  |  |  | O | O | O | O | O |
| 8 |  |  |  |  |  |  |  | O | O | O | O |
| 9 |  |  |  |  |  |  |  |  | O | O | O |
| 10 |  |  |  |  |  |  |  |  |  | O | O |
| 11 |  |  |  |  |  |  |  |  |  |  | O |

### Posterior/A-P Width

|  | 2 | 3 | 4 | 5 | 6 | 7 | 8 | 9 | 10 | 11 | 12 |
| --- | --- | --- | --- | --- | --- | --- | --- | --- | --- | --- | --- |
| 1 | O | O | O | O | O | O | O | O | O | O | O |
| 2 |  | O | O | O | O | O | O | O | O | O | O |
| 3 |  |  | O | O | O | O | O | O | O | O | O |
| 4 |  |  |  | O | O | O | O | O | O | O | O |
| 5 |  |  |  |  | O | O | O | O | O | O | O |
| 6 |  |  |  |  |  | O | O | O | O | O | O |
| 7 |  |  |  |  |  |  | O | O | O | O | O |
| 8 |  |  |  |  |  |  |  | O | O | O | O |
| 9 |  |  |  |  |  |  |  |  | O | O | O |
| 10 |  |  |  |  |  |  |  |  |  | O | O |
| 11 |  |  |  |  |  |  |  |  |  |  | O |

### Lateral/Lateral Width

|  | 2 | 3 | 4 | 5 | 6 | 7 | 8 | 9 | 10 | 11 | 12 |
| --- | --- | --- | --- | --- | --- | --- | --- | --- | --- | --- | --- |
| 1 | O | O | O | O | O | O | O | O | O | O | O |
| 2 |  | O | O | O | O | O | O | O | O | O | O |
| 3 |  |  | O | O | O | O | O | O | O | O | O |
| 4 |  |  |  | O | O | O | O | O | O | O | O |
| 5 |  |  |  |  | O | O | O | O | O | O | O |
| 6 |  |  |  |  |  | O | O | O | O | O | O |
| 7 |  |  |  |  |  |  | O | O | O | O | O |
| 8 |  |  |  |  |  |  |  | O | O | O | O |
| 9 |  |  |  |  |  |  |  |  | O | O | O |
| 10 |  |  |  |  |  |  |  |  |  | O | O |
| 11 |  |  |  |  |  |  |  |  |  |  | O |
